# Human corticospinal tract lateralization at the height of the internal capsule is not related to handedness

**DOI:** 10.1038/s41598-025-19580-9

**Published:** 2025-09-24

**Authors:** M. Domin, R. Lindenberg, M. Lotze

**Affiliations:** 1 Functional Imaging Unit, Institute of Diagnostic Radiology and Neuroradiology, University Medicine Greifswald, Greifswald, Germany; 2 Department of the History, Philosophy and Ethics of Medicine, Medical Faculty, Department of Neurology, Heinrich- Heine University Duesseldorf, ZAR Berlin, Berlin, Germany

**Keywords:** Corticospinal tract, Handedness, Fractional anisotropy, DTI, Stroke, Human connectome project, Stroke, Sensorimotor processing

## Abstract

Evaluating the integrity of the corticospinal tract at the height of the posterior limb of the internal capsule with a lateralization index has been applied to predict upper limb motor recovery after stroke in numerous diffusion tensor imaging studies. When comparing patient groups with healthy controls in this context, matching for age and gender is generally recommended. Since a generalized lateralization of diffusion strength to the dominant left hemisphere has been reported, it can be argued that handedness should also be accounted for. To address this question, we used the Human Connectome Project data set containing 1,065 diffusion-weighted MRI sets as well as information on handedness as defined by the Edinburgh Handedness Inventory. We hypothesized that handedness might be related to diffusion strength of the corticospinal tract. As commonly employed, we extracted fractional anisotropy values at the level of the internal capsule to calculate a laterality index. Contrary to our hypothesis, we found no association between corticospinal tract diffusion strength and handedness. We conclude that for handedness, no balancing between patient and control groups is needed when comparing corticospinal tract diffusivity parameters.

## Introduction

The question whether handedness, as defined by the Edinburgh Handedness Inventory^1^is related to lateralization of corticospinal tract strength in humans has been addressed in several studies over the past decades. Kertesz and Geschwind^2^ investigated the relation between cords in the corticospinal tract (CST) crossing to the opposite lateral column and found no relation to handedness. However, those post mortem analyses were methodologically quite demanding restricting sample sizes (overview in^3^. With the advent of methods such as multi-directional diffusion imaging (MDDI), white matter quantification became possible in vivo. It yielded similar results compared to earlier post mortem analyses (humans: e.g., ^4^; rodents: e.g., ^5^. Diffusion tensor imaging (DTI) became widely available, and with more studies being conducted the need to establish gold standard methods to ensure reproducibility of DTI quantification was apparent. Besides the above studies in healthy subjects, DTI-derived features of the CST were also used for correlations between structure and motor function as well as for outcome predictions in patients after stroke^6^. In addition, CST integrity had been assessed as a relevant biomarker for motor recovery following stroke by the Stroke Recovery and Rehabilitation Roundtable in 2017^7^. One of the most commonly used parameters in this context is the fractional anisotropy (FA) laterality index (LI) of the posterior limb of the internal capsule (PLIC). FA is a scalar value that ranges between 0 and 1 with higher values reflecting more restricted diffusion. Thus, for a given tract it can serve as a surrogate marker of its structural integrity. It is especially valuable when no major change of direction occurs, and no other fibers cross. Now a laterality index can be calculated between the average FA values of the PLICs of both hemispheres, ranging from − 1 to + 1, where 0 would be a balanced laterality. For this purpose, the PLIC is well suited because it can be easily identified on axial FA images as it primarily consists of densely packed CST fibers that mostly run in a vertical direction. Among its use in other studies, LI_PLIC allowed to differentiate poor from moderate upper limb motor recovery after stroke in outcome prediction algorithms^8^. Specifically, the authors demonstrate that, in the absence of motor evoked potentials, recovery potential was lower with lower CST integrity. This algorithm had been effectively used to balance treatment groups for testing intervention effects^9^ and showed robust effects predicting upper limb function following stroke in different study groups (e.g., ^10–12^. Park and colleagues^13^ compared different methods for quantifying diffusion parameters of the CST and demonstrated that LI_PLIC is a valid, robust and sensitive method.

In an early DWI-quantification study, mean diffusivity (MD) and FA of the PLIC were compared in 30 left-handed and 30 right-handed healthy subjects^14^. No interaction of handedness or PLIC lateralization was found for either parameter. However, both a relatively low number of participants (from today’s perspective) and the technical developments in diffusion weighted imaging and DTI-quantification over the past years call for a renewed dedication to this topic.

It has been shown repeatedly that age has an impact on CST lateralization^15,16^. Thus, balancing age between study groups is currently routine especially when investigating upper limb function after stroke (e.g., ^17^. Furthermore, gender appears to impact on both DTI-derived parameters of white matter lateralization and upper limb impairment (for infants^18^; for adolescents^19^, although results are somewhat heterogeneous (e.g., ^20^. 

In addition, a reported lateralization of white matter diffusion properties makes it necessary to control for hemisphere side in between-group comparisons^14^. Therefore, when studying patients with unilateral lesions, the affected hemisphere needs to be balanced also in the healthy control group.

In order to answer whether handedness might have an impact on lateralization of DWI-parameters in a sufficiently powered group with current state of the art imaging procedures, we analyzed a large number of data sets of healthy participants with extraordinary diffusion imaging quality provided by the Human Connectome Project (HCP). We were thus able to include a sample of 1065 healthy young volunteers with an almost balanced gender distribution and a sufficient proportion of left-handers (*n* = 72).

## Methods

### Data description

Diffusion-weighted scans were obtained by the HCP (http://www.cmrr.umn.edu/multiband). The data from the HCP young adult study, comprising 1,200 subject data sets, were used in this analysis. Of these, 1,065 contained valid diffusion-weighted MRIs (dMRI). The subjects’ ages ranged from 22 to 35 years, with a mean age of 28.75 ± 3.67 years. Of the subjects, 575 were female and 490 were male. As defined by Oldfield^1^ for the EHI, types of handedness can be selected by imposing thresholds; left-handedness: EHI ≤ -40 (*n* = 72), ambidexterity: EHI between − 40 and + 40 (*n* = 67) and right-handedness: EHI ≥ + 40 (*n* = 926).

The diffusion MRI data set consisted of three distinct gradient tables, including 89, 90 and 91 diffusion weighting directions, respectively. Each set was acquired on a single occasion with right-to-left and left-to-right phase encoding polarities, resulting in 6 acquisitions per participant. Six b = 0 volumes were distributed throughout and acquired during each of the six runs. The diffusion weighting consisted of three shells of b = 1000, 2000, and 3000 s/mm², interspersed with an approximately equal number of acquisitions on each shell within each run. The diffusion directions were obtained using a toolbox, available from INRIA, that returns directions distributed uniformly in multiple q-space shells. The directions are optimized in such a way that every subset of the first M directions is also isotropic^21^.

The following global scan parameters were employed for the multiband diffusion sequence: voxel size = 1.25 mm³, slices = 111, TR = 5520, TE = 89.5. This resulted in a scan time of 9:41 min for each acquisition. For a comprehensive overview of the sequences employed, please refer to the HCP Reference Manual (WU-Minn HCP 1200 Subjects Release: Reference Manual –> Appendix I - Protocol Guidance and HCP Session Protocols –> Diffusion Session).

## Preprocessing

FMRIB’s Software Library 5 (FSL 5)^22^ was employed to rectify distortions resulting from susceptibility (FSL TOPUP) and eddy-current-induced artifacts (FSL EDDY). In contrast to susceptibility-related artifacts, which are constant in a spatial manner throughout the measurement, eddy-current-induced distortions are unique to each diffusion-weighted volume^23^. The estimation and correction of susceptibility-induced distortions capitalize on the complementary information inherent in pairs of diffusion images acquired with reversed phase-encoding (PE) directions. Reversing the phase-encoding direction negates the effect of susceptibility-induced distortions. The combination of both images allows for estimation of an off-resonance field. In the HCP processing pipeline, estimation is the sole stage that occurs prior to the subsequent stage, which estimates eddy-current-induced distortions and motion-related artifacts. In this step, the complementary information from two nearly opposing diffusion gradient directions was utilized, as it was anticipated that they would exhibit nearly opposite eddy-current-induced distortions, whereas susceptibility-induced distortions would remain unchanged. Utilizing the previously constructed field map as a foundation, these data were fed into a Gaussian Process predictor, which was employed to additionally estimate the eddy-current induced field and the subject motion for each diffusion-MRI volume. The estimated field maps were then combined and used to correct the volumes in a single resampling step and spline interpolation^24^.

## Diffusion tensor and Spatial normalization

FSL’s dtifit was employed to calculate the diffusion tensor. The resulting tensor images (Eigenvector and Eigenvalue) were fed into DTI-TK, a specialized tool for optimized registration and spatial normalization of diffusion tensor data. DTI-TK uses the whole tensor matrix at each voxel to calculate a correspondence between images. Additionally, an iterative, sample-based average diffusion tensor image (sample template) was computed in order to further improve registration between subjects. The resulting template image was registered to the MNI152 ICBM 6th gen. 2009c asymmetric nonlinear template so as to obtain a combined transformation from individual subject space to MNI template space via the sample template, which could then be used to transform each diffusion tensor image into MNI template space in a single interpolation step. Fractional anisotropy images were then calculated and used for further assessment.

## Extraction of fractional anisotropy

**Fig. 1:**
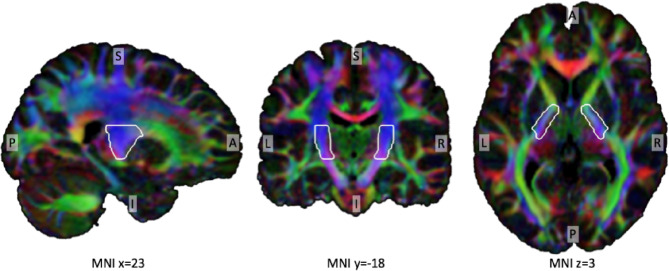
For illustrative purpose, the region-of-interest of the posterior limb of the internal capsule (white border), overlayed onto a MNI DTI template. The tensor’s first Eigenvector is shown, the colors red, green and blue assigned to each vector component x, y and z, respectively.

Regions-of-interest of the posterior limb of the internal capsule for both hemispheres (Left, Right) were selected from the ICBM-DTI-81 white-matter labels atlas^25^ (see Fig. 1). Average fractional anisotropy was extracted for both regions of interest (ROI) in every subject (FA_PLIC_L, FA_PLIC_R) and the laterality index for both ROIs was calculated by

$$\:LI\_FA\_PLIC=\frac{FA\_PLIC\_L\:-\:FA\_PLIC\_R}{FA\_PLIC\_L\:+\:FA\_PLIC\_R}$$

## Statistics

The statistical analysis of the fractional anisotropy data was conducted with IBM SPSS Statistics (v. 29.0.1.1). Differences were determined using paired and independent samples t-tests or their non-parametric counterparts, and to calculate the relation between two dependent variables, Pearson’s bivariate correlation or Spearman´s ρ were applied, all depending on Kolmogorov-Smirnoff’s test for normality.

## Results

The mean age for the 1065 participants of the HCP sample included in our study was 28.8 ± 3.7 years. The sample comprised more females than males (54% vs. 46%) as well as 6.8% (*n* = 72) left-handers and 6.3% (*n* = 67) ambidextroushanders.

The fractional anisotropy of the posterior limb of the internal capsule showed a strong difference between hemispheres for all participants in a Wilcoxon Signed Ranks test: *Z* = -27.47, *p* < 0.001, Cohen’s d = 3.117, indicating a clear lateralization to the right hemisphere. When separating the data into sub-groups, Wilcoxon Signed Ranks tests showed a strong FA laterality to the right hemisphere in all subgroups, i.e., the difference between the left and the right hemisphere, in all cases: (a) for the EHI ≤ -40 (left-handers; *n* = 72): *Z* = -7.13, *p* < 0.001, Cohen’s d = 3.1, (b) for ambidextrous handers (between EHI − 40 and 40 ; *n* = 67): *Z* = -6.83, *p* < 0.001, Cohen’s d = 3.028 and (c) right handers (EHI ≥ 40; *n* = 926): *Z* = -25.64, *p* < 0.001, Cohen’s d = 3.129.

**Fig. 2:**
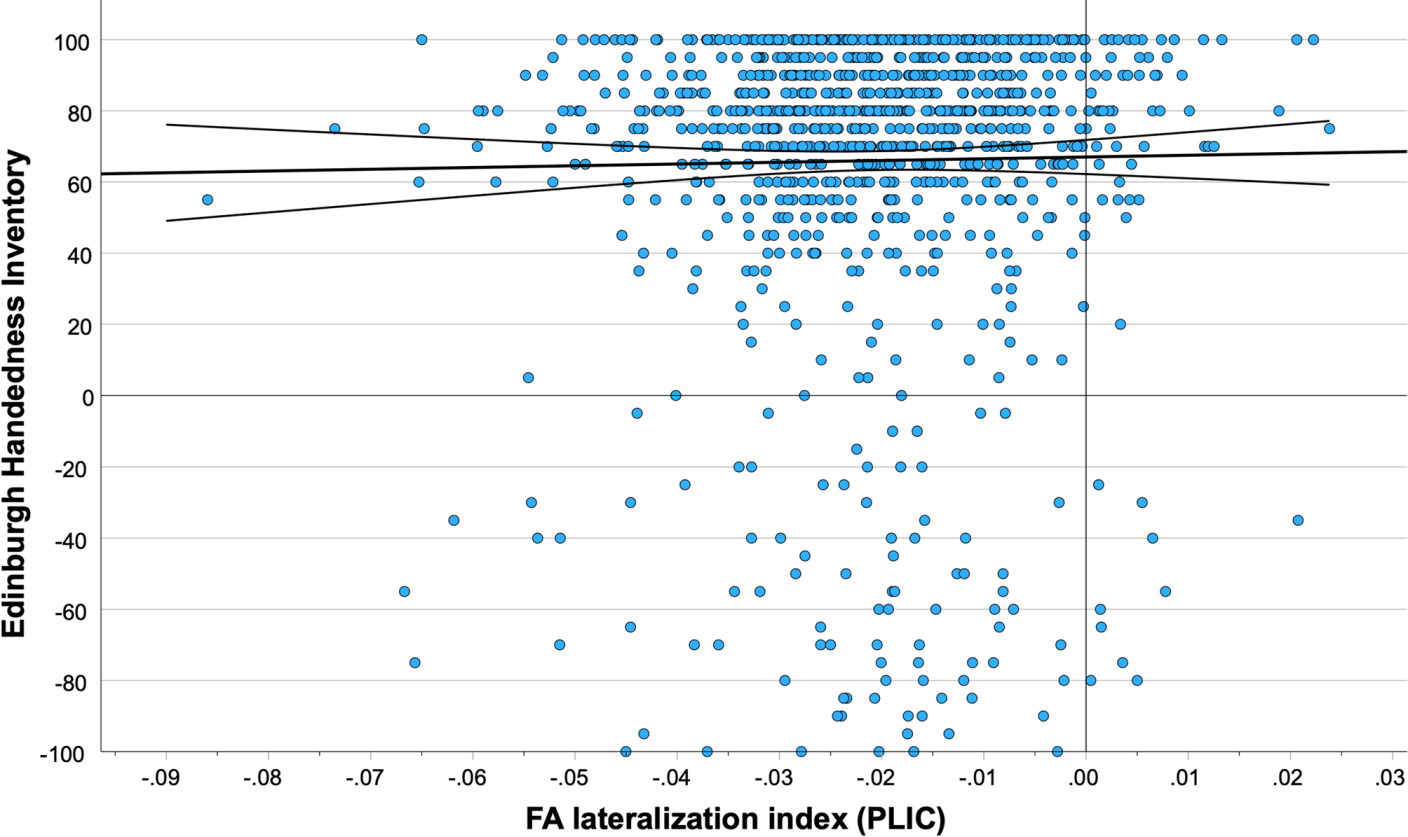
Plot of FA lateralization index, assessed in the posterior limb of the internal capsule and Edinburgh Handedness Score. We found no relevant association between both measures (ρ(1065) = 0.034, *p* = 0.261). Trendline and confidence intervals provided in black.

Handedness (as assessed by the Edinburgh Handedness Inventory, EHI) was not related to LI_FA_PLIC: Spearman´s ρ revealed no relevant association (ρ(1065) = 0.034, *p* = 0.261; see Fig. 2).

When separated into two groups, each containing only left-handers and right-handers, respectively, no associations could be found between LI_FA_PLIC and EHI. For left-handers: (ρ(72) = -0.031, *p* = 0.797) and for right-handers: (ρ(926) = 0.061, *p* = 0.063). Furthermore, an Independent-Samples Mann-Whitney U Test showed no statistically relevant differences between both groups with regards to LI_FA_PLIC: U = 31045.00, Z=-0.972, *p* = 0.331. As age is usually a strong confounder in regards to fractional anisotropy, Spearman’s ρ was calculated for the association between age and LI_FA_PLIC: ρ(1065) = 0.094, *p* = 0.002.

A Wilcoxon Signed Ranks test revealed a statistically significant difference in LI_FA_PLIC for male and female participants: *Z* = -4.89, *p* < 0.001, Cohen’s d = 0.303.

## Discussion

Based on optimized diffusion imaging in a large number of participants we demonstrated that handedness (as assessed by the EHI; Oldfield, 1971) was not related to LI_FA_PLIC. This finding is underlining results from prior investigations in smaller sample sizes using different white matter quantification methods such as post mortem histopathology^2^ or DTI^14,15^. Methodological developments in diffusion imaging and data analysis as well as readily available large data sets enabled us to test for potential smaller effect sizes that couldn’t have been detected previously. As such, Volz et al.^26^ investigated the number of fiber orientations per voxel providing a probabilistic white matter atlas of distinct fiber directions (one, two or three). Regarding LI_FA of the CST they found an asymmetry towards the left hemisphere for one-directional voxels, and towards the right hemisphere for multi-directional voxels, respectively. On a side note, they report similar results for their group of 630 healthy subjects and a sub-sample of 49 left-handers taken from the HCP, suggesting that hemispheric FA differences in the CST were not driven by handedness. In contrast, we placed our ROI at the PLIC level, which contained almost exclusively one-directional voxels. Hence, we didn’t have to account for multi-directionality. Taken together, we were able to replicate the results by Volz et al. in an even larger cohort. Furthermore, our findings complement those results by focusing on the commonly used approach of placing the ROI at the PLIC level.

If there had been an association between handedness and LI_FA_PLIC, it would be necessary to balance participant groups accordingly. Otherwise, accounting for gender, hemisphere (right and left) and age should be sufficient.

Especially age has a considerable impact on LI_FA_PLIC since lateralization is increasing during childhood but decreasing in late adulthood^16^the latter being the most probable time window for a stroke^27^. In a recent review, Budisavlevic et al.^28^ formulated that although handedness is related to asymmetry of intrahemispheric pathways such as the superior longitudinal fasciculus, the CST as a projection tract shows no such associations.

It needs to be taken into account that methods for assessing dexterity and handedness are a crucial issue in the context of this study. Given a long history of criticizing shortcomings of the EHI, it has been modified^29^shortened and reformulated^30^. However, most large cohorts – such as the HCP – still use the original version. In this respect we were bound by this questionnaire although it could be questioned whether it corresponds to biologically relevant data of dexterity. Büchel and others^31^using voxel-based analysis of DWI reported an effect of hand preference on the white matter quantification of the precentral gyrus. FA was higher in the left compared to the right hemisphere in right-handers and vice versa in left-handers. More differentiated assessment and quantification of hand dexterity in musicians^32^ or by movement kinematics of circle drawing showed associations with white matter lateralization in the CST^33^. The later study demonstrated that linear regression models predict circle drawing laterality with right–left FA and MD asymmetry in a ROI covering the corticospinal tract. However, other studies failed to demonstrate those associations, so it can be assumed that lateralization seems to be highly task dependent^34^. In the context of post stroke studies, an assessment of handedness and dexterity based on execution of motor acts becomes even more complicated due to impaired motor function. In contrast, questionnaires such as the EHI can be used to retrospectively assess lateralization of certain functions independent of potential post-stroke impairment. Taken together, while considerable issues with the EHI need to be considered, it remains the most widely used tool to quantify handedness.

## Limitations and further research

For the HCP participants´ age ranged from 22 to 35 years, with a mean age of 28.75 ± 3.67 years. Since age is an important factor for LI_FA_PLIC, a population in the same age range as stroke patients might be quantified in the next investigation on that topic. Although multiple statistical testing occurred with similar variables, most tests, especially about hemispherical laterality, showed consistent results, even for sub-groups. We therefore decided not to correct for multiple comparisons^35,36^. It is also necessary to mention the low percentage of left-handed participants in the HCP-study which might limit the significance of this study.

Spatial normalization of human brain data is a highly complex process, distorting the brain’s topology in order to obtain an as good as possible spatial congruence with a brain template. The calculated distortions highly depend on the used algorithms, the comparison metrics during the process and the compute time allowed for reaching a convergence. In our case DTI-TK was used, an algorithm that incorporates the whole diffusion tensor, increasing the precision especially in the white matter. This makes it harder to compare our results with studies using inferior procedures. In this context a second issue has to be mentioned, the use of generic atlases or ROIs. Results generated by means of generic atlases or ROIs are always highly dependent on the original data they were created from and the spatial normalization for a proper fit between ROI and the data. As this study uses atlas-based ROIs (as most research groups and clinicians would do), results have to be interpreted with caution.

## Conclusions

Our results show that balancing groups (e.g., healthy volunteers and stroke patients) with regard to hemisphere, sex and is sufficient when investigating LI_FA_PLIC. Regarding handedness, no balancing between groups is needed.

## Data Availability

The data that support the findings of this study are available from the corresponding author upon request. The Human connectome data are available for research purpose.
